# Exploring antenatal care utilization and intimate partner violence in Benin - are lives at stake?

**DOI:** 10.1186/s12889-021-10884-9

**Published:** 2021-04-30

**Authors:** Dina Idriss-Wheeler, Sanni Yaya

**Affiliations:** 1 Interdisciplinary School of Health Sciences, University of Ottawa, Ottawa, Canada; 2 School of International Development and Global Studies, Faculty of Social Sciences, University of Ottawa, 120 University Private, Ottawa, ON K1N 6N5 Canada; 3 The George Institute for Global Health, Imperial College London, London, UK

**Keywords:** Antenatal care (ANC) utilization, Intimate partner violence (IPV), Maternal health, Demographic health survey (DHS), Benin, Cross-sectional study

## Abstract

**Background:**

The republic of Benin ranks in the bottom third of countries recently assessed for ANC coverage and its Ministry of Family and National Solidarity (2009) reported close to 70% of Beninese women suffered abuse at least once in their lifetime. Utilization of antenatal care (ANC) services is key to positive health outcomes for both mother and infant. This study examined the impact of intimate partner violence (IPV) on the utilization of ANC services in Benin using both the basic 4 visit model (ANC-4) and the updated WHO recommended 8-visit model (ANC-8).

**Methods:**

Data used for this study were collected from the nationally representative 2017–2018 Benin Demographic Health Survey (BDHS) on ever-partnered women aged 15–49 who had completed both reproductive maternal health and domestic violence modules of the survey. Descriptive statistics and multivariate logistic regression analysis were performed to determine significant factors associated with ANC utilization in Benin.

**Results:**

Over 40% of the women (*n* = 3084) reported experience of IPV in their lifetime. Findings revealed that women who ever experienced IPV (OR 0.753, 95% CI: 0.628–0.901; *p* = 0.002) had 25% less odds of accessing the basic four ANC visits. IPV was not found to be a factor in accessing at least eight ANC visits. With increasing number of children, there was less likelihood of accessing at least four and at least eight visits. Being in the richest quintile (OR 5.490, 95% CI 3.907–7.714; *p* < 0.000 for ANC-4; OR, 5.781, 95% CI: 3.208–10.41; *p* < 0.000), making decisions on household and health care (OR 1.279, 95% CI: 1.042–1.569 for ANC-4; OR, 1.724; 95% CI: 1.170–2.540; *p* = 0.006 for ANC-8), and getting paid cash for work increased the chances of utilizing ANC-four (OR 1.451, 95% CI: 1.122–1.876; 0.005) but not for ANC-eight. Belonging to the Muslim faith decreased the odds of ANC utilization compared to all other religions.

**Conclusion:**

This work revealed key areas for maternal health policy makers and service providers in Benin to appropriately plan effective policies (i.e., alleviate poverty; equitable health services access; cultural sensitivity) and necessary interventions (i.e. ANC education, IPV prevention, paid employment, alcohol cessation) to increase utilization of ANC.

**Supplementary Information:**

The online version contains supplementary material available at 10.1186/s12889-021-10884-9.

## Background

Antenatal care (ANC) is provided by skilled health professionals to pregnant women and girls to ensure optimal health conditions for both mother and foetus during pregnancy [1]. For a positive pregnancy experience, the following components are essential to ANC: risk identification, prevention and management of pregnancy-related or concurrent diseases, and health education and health promotion [1]. Studies have shown that a higher frequency of ANC reduced likelihood of still births and other morbidities because of early detection and management of complications [2].

The maternal mortality rates remain unacceptably high in sub-Saharan Africa (SSA). The most recent figures estimate 534 maternal deaths per 100,000 live births in SSA compared to 211 maternal deaths per 100,000 live births globally [3]. The WHO has been working to improve quality of care and increase ANC coverage in low- and middle-income countries (LMICs) for the past two decades [1]. Research on ANC underutilization continues to emerge in low- and middle-income countries (LMICs) indicating continued negative outcomes for mothers and newborns [4, 5]. Understanding what factors could be affecting underutilization is important, especially if the goal of LMICs is to create policies, programs and interventions to increase ANC utilization. Factors affecting utilization of ANC among pregnant women have been identified; namely, maternal age, number of living children, education, place of residence, employment and source of income, decision making authority (autonomy), socio-demographic and economic characteristics of partner or husband, religion and ethnicity [6]. One emerging area of interest is the intersection between ANC utilization and intimate partner violence (IPV) where both the lives of the mother and child are at risk of not only direct physical harm, but also the psychological and emotional consequences of the perpetrated violence.

The WHO defines IPV as any behaviour within an intimate relationship that causes physical, psychological or sexual harm to those in the partnership [7]. Controlling behaviours that lead to isolation from family and friends, monitoring movements, restricting access to financial resources, employment, education or medical care are also key considerations of violence perpetrated against women [7]. IPV is linked to a multitude of poor health outcomes for women, one of which is sexual and reproductive health [7, 8]. According to the WHO, on average, one in three ever partnered women around the world has experienced IPV in her lifetime [7]. The story is dismal for countries in SSA where high levels of IPV affect 36% of the women [9].

A recent meta-analysis and systematic review of LMICs demonstrated the effect of IPV on utilization of ANC, suggesting that women who experienced IPV had 25% decreased odds of using adequate ANC than women who did not [10]. Other country specific studies have shown no association between IPV and ANC [4]. With mixed findings across differing cultural contexts, it is important to continue to investigate factors that can affect utilization, particularly in regions with high incidences of IPV and low utilization of ANC.

## Methods

### Study setting

The republic of Benin is located in West Sub-Saharan Africa and reports a maternal mortality ratio of 400 per 100,000 live births [11]. Benin ranks in the bottom third of countries recently assessed for ANC coverage and in 2009, the Ministry of family and National Solidarity reported 69% of Beninese women suffered abuse at least once in their lifetime [9, 12–14]. More recently, in 2019, the UN Women’s Global Database on Violence against Women reported a 24% prevalence of lifetime physical and/or sexual IPV in Benin, with no report of emotional of psychological IPV [15]. Cross-sectional studies from previous Demographic and Health Surveys (DHS) found close to 40% of mothers received less than 4 ANC visits or never attended services, suggesting household wealth and female education as key factors [16, 17]. With the existing issue of high maternal mortality rates, low utilization of necessary antenatal care services and exposure to IPV in Benin, the aim of our study was to examine the impact of intimate partner violence on utilization of ANC services by women in Benin, where utilization is suboptimal and rates of violence against women are high.

### Data source

The data used for this study were collected from the 2017–2018 Benin Demographic Health Survey (BDHS) [18]. This nationally representative population and health survey was conducted in Benin by the Ministry of Planning and Development and the National Institute of Statistics and Economic Analysis (INSAE) in collaboration with the Ministry of Health, Centre for Parasitology & Cardiology Laboratory of the National Hospital and the Permanent Secretariat of the Food and Nutrition Council. Technical support was provided by ICF International and funding support by UNAIDS, UNICEF, UFPA and the World Bank [18].

### Sampling

The sampling process is described in detail in the 2017–2018 BDHS report; results are representative of the country as a whole and of urban and rural areas separately for 12 administrative departments in Benin [18]. A total of 14,435 households were selected, 16,233 females aged 15–49 were eligible for interviews and 15, 928 females were actually interviewed (98.1% response rate). A total of 3317 women were eligible for our study (i.e. answered the ANC question and the domestic violence module). After completing listwise deletion, the final sample size was 3084 Beninese women aged 15–49.

### Variable selection and measurement

The outcome variable was ANC utilization by ever-partnered women aged 15–49 in Benin in 2017–2018. Although the WHO revised the recommendation from at least a four-visit to at least an eight-visit model in 2016, only 54% of the women in sub-Saharan Africa reached four ANC visits, and for the few countries which reached the recommended eight ANC visits, the quality of health services and distribution across the region was an issue [1, 19]. In order to adequately assess the utilization of ANC in Benin, we used both the basic antenatal care coverage model of at least four-visits outlined by the WHO Global health Observatory [20] and the recently recommended at least 8 visit model. The ratio variable ANC-4 was recoded into a binary level measurement; 0 for “0 to 3” visits and 1 for “4 or more” ANC visits during last pregnancy for the basic model. For the updated at least 8 visit model, the variable (ANC-8) was recoded as 0 for “0 to 7” visits and 1 for “8 or more’ ANC visits during the last pregnancy.

The main independent variable was intimate partner violence (IPV), which was recoded to a dichotomous form. IPV included 10 questions on emotional, sexual and physical violence experienced by the women (i.e. ever: pushed, slapped, twist arm/pull hair, punch/hit with something, kick/drag/beat, choke/burn, threaten with knife/gun/weapon, physically force sexual intercourse, physically force sexual acts, force sexual acts with threats) [18]. Each question was recoded into a dummy variable; if the answer was “often”, “sometimes” (which pertain to violence in the last 12 months) and “yes, but not in the last 12 months” for any of the questions on emotional, sexual or physical violence, it was coded *yes = 1* and if the answer was *never* then it was *no = 0*. The question dummy variables were summed to generate a summed dummy variable that provided a ratio level variable accounting for women who never experienced IPV and women who ever experienced at least one form of violence (answered yes to one or more of the questions). These were then recoded into a binary variable of *ever experienced IPV* where *never = 0* and *ever = 1 or more.*

The exploratory variables were used as controls to inform the predictive models and outcome of utilization of ANC services in Benin. Decision making was created using four questions on participation in household decision making (i.e. person who usually decides on: respondent’s health, large household purchases, visits to family/relatives, what to do with money husband earns) [18]. Decisions made by “respondent alone” or “respondent and husband/partner” were recoded as 1, *decision making*. The decisions made by ‘husband/partner alone”, “someone else”, or “other” were recoded as 0, *no decision making*. A summed dummy variable provided a count which was recoded to generate a dichotomous form of *no decision making* (participated in no decisions = 0) and *some decision making* (participate in one or more decisions = 1).

Age of woman was divided into three sub-groups (15–24, 25–34, and 35–49); the youngest subgroup was the reference for regression analysis. Two categories existed for place of residence: rurality (recoded as rural = 1, urban = 0) and urbanicity (recoded as urban = 1, rural = 0). The DHS wealth index (poorest, poor, middle, rich, richest) was used and the poorest quintile was the reference group in regression analysis. In DHS, the wealth index is a composite measure of cumulative living standard of a household based on ownership of select assets (i.e. televisions, bicycles, cars, dwelling characteristics, type of drinking water source, toilet and sanitation facilities) [18].

The level of education for respondent and partner were divided into: (i) no education (reference group) and included women or partners with no education and incomplete primary education; (ii) primary education which included women or partners who had primary and incomplete secondary education; and (iii) secondary or higher which included individuals with secondary or higher education. The DHS asks the female participants if they are ‘working’ and this was recoded as 1 for “yes” and 0 for “no”. For the husband or partner employment, if they “did not work” it was recoded as 0, *not employed,* and if they had employment (professional, sales, agricultural, services, skilled manual, other unclassified or don’t know), it was recoded as 1, *employed*. The questionnaire also provided insight into husband or partner’s alcohol drinking and answers were recoded as 1 for “yes” (drinks alcohol) and “no” (does not drink alcohol).

Religion was subdivided into Muslim, Christian, Catholic, Traditional, Other or no religion; Muslim was the reference group in the regression analysis based on previous studies looking at religion and ANC [21]. The number of children was divided into one child (reference group), two to four children and five or more children. Finally, attitude toward wife beating was included as an exploratory variable based on empirical evidence in Benin [14]. The five questions regarding ‘beating being justified if the wife goes out without telling husband, wife neglects the children, wife argues with husband, wife refuses to have sex with husband, wife burns food [18] were recoded 1 if the answer was “yes” to any of the questions. If participants answered “no” or “I don’t know”, it was recoded as 0. A summed dummy variable provided a count which was recoded to generate a dichotomous form of *justified* = 1 and *not justified* = 0. Detailed syntax for coding structure and running the analysis is available (see Additional file 1).

### Statistical analysis

The complex sample design of the DHS was accounted for in significance testing with sample weights using STATA’s *svyset and svy* commands. Statistical significance between groups were estimated using bivariate regression tests for women who ever experienced IPV and those who never experienced IPV. To investigate the relationship between IPV and ANC utilization, multiple logistic regression was carried out with all of the covariates included in the model. The data were analyzed using the software package STATA version 16 [22].

## Results

### Descriptive statistics

Of the 3084 women in the sample, close to 56% (*n* = 1715) accessed at least four ANC visits and only 10% (*n* = 312) accessed the WHO recommended eight visits. Additionally, there were 41.54% of the women who *ever* experienced IPV (*n* = 1281) and 58.46% (*n* = 1803) who *never* experienced IPV. As shown in Table 1, a statistically significant lower proportion of Beninese women who ever experienced IPV used ANC-4 services (51.85%) in comparison to women who never experienced IPV (60.14%). Similarly, a significantly lower proportion of the women who experienced violence (8.68%) accessed ANC-8 compared to women who had not experienced violence (11.26%). These findings suggest that women who never experienced IPV had made more decisions regarding healthcare, household purchases or spending and visiting family relatives. Significantly fewer women who ever experienced IPV (20.75%) compared to those who did not (25.83%) were 15–24 years old but more women aged 25–34 had experienced violence (55.21%) than women who had not (50.70%).

**Table 1:** Descriptive Statistics by Women who Ever Experienced Intimate Partner Violence (IPV) in Benin (IDSB-V, 2017–2018)

	Ever Experienced IPV	95% Confidence Interval	Never Experienced IPV	95% Confidence Interval
Antenatal Care - 4 Visits	51.853%***	[48.805, 54.900]	60.137%	[57.400, 62.880]
Antenatal Care - 8 Visits	8.679%*	[07.015, 10.342]	11.263%	[09.545, 12.981]
***Respondent Characteristics***				
Empowerment				
Decision Making	73.172%*	[69.980, 76.368]	76.820%	[74.523, 79.118]
Age (in years)				
15–24	20.753%**	[18.385, 23.122]	25.831%	[23.713, 27.950]
25–34	55.208%*	[52.306, 58.110]	50.700%	[48.210, 53.189]
35–49	24.039%	[21.624, 26.454]	23.469%	[21.391, 25.547]
Type of Residence				
Rural	63.359%**	[59.853, 66.864]	57.393%	[54.337, 60.449]
Urban	36.641%**	[33.136, 40.147]	42.607%	[39.551, 45.663]
Education				
None	85.920%***	[83.734, 88.105]	79.302%	[77.125, 81.480]
Primary	13.102%**	[10.993, 15.211]	18.863%	[16.792, 20.935]
Secondary or Higher	00.978%*	[00.431, 01.525]	01.834%	[01.117, 02.494]
Wealth Index				
Poorest	19.006%	[15.996,22.016]	18.811%	[16.263, 21.358]
Poorer	21.245%*	[18.415,24.075]	17.205%	[15.286, 19.124]
Middle	21.488%	[18.832, 24.143]	20.434%	[18.198, 22.671]
Rich	20.732%	[17.967, 23.497]	21.194%	[18.900, 23.488]
Richest	17.530%**	[14.909, 20.150]	22.356%	[19.771, 24.941]
Employment Status				
Working	86.111%***	[83.995, 88.228]	80.980%	[78.867, 83.093]
Paid	74.743%	[71.871, 77.615]	71.867%	[69.328, 74.406]
Number of Children				
1 child	11.378%***	[09.464, 13.292]	18.681%	[16.662, 20.700]
2–4 children	54.994%	[52.067, 57.922]	52.536%	[50.015, 55.056]
5 or more children	33.627%**	[30.802, 36.452]	28.784%	[26.492, 31.078]
Religion				
Muslim	26.246%**	[23.203, 29.289]	31.884%	[29.007, 34.761]
Catholic	20.689%	[17.769, 23.609]	22.387%	[19.925, 24.849]
Christian	24.571%	[21.743, 27.400]	23.512%	[21.134, 25.890]
Traditional	11.558%*	[09.351, 13.766]	8.710%	[07.007, 10.412]
Other	10.571%	[08.587, 12.555]	10.137%	[08.407, 11.868]
No Religion	06.365%***	[04.735, 07.995]	3.370%	[02.443, 04.296]
Attitude on Wife Beating				
Justified	34.606%%	[31.288, 37.924]	31.347%	[28.598, 34.096]
***Husband or Partner Characteristics***				
Education				
None	72.203%**	[69.373, 75.032]	66.848%	[64.180, 69.516]
Primary	18.981%	[16.611, 21.351]	20.225%	[18.112, 22.338]
Secondary or Higher	03.949%***	[02.778, 05.121]	09.535%	[07.941, 11.129]
Unknown	04.867%	[03.462, 06.272]	03.392%	[02.153, 04.369]
Employment Status				
Working	99.051%	[98.536, 99.565]	98.671%	[98.160, 99.182]
Drinks Alcohol	41.943%***	[38.811, 45.075]	17.481%	[15.460, 19.501]
n	1281		1803	

* *p* ≤ 0.05, ** *p* ≤ 0.01, *** *p* ≤ 0.001; Total sample is 3084

Significantly more women who ever experienced violence had no education, belonged to the poorer quintile, were working and lived in rural regions of Benin. In terms of wealth, more of the women who had not experienced violence (22.36%) were in the richest quintile compared to women who ever experienced violence (17.53%). There were more women who had not experienced violence who had one child (18.68%) compared to those who had experienced violence and had one child (11.38%), yet fewer women who had no experience of violence had five or more children (28.78%) compared to women who had experienced violence (33.63%). More of the Muslim women had not experienced violence (31.88%) compared to those who had (26.25%). No significant difference was found between the two groups in terms of attitudes toward wife beating.

A higher proportion of women who had not experienced violence had more educated partners, particularly with secondary education or higher. A staggering percentage of women who had experienced any form of violence had husbands or partners who drank alcohol compared to women who never experienced violence. No difference was present between the groups in terms of husband or partners’ employment**.**

## Multivariate logistic regression models

Table 2 presents the multivariate model indicating that being exposed to IPV decreased the likelihood of ANC-4 utilization. A Beninese woman’s odds of utilizing ANC-4, if she ever experienced IPV, were 25% (OR, 0.753; 95% CI: 0.628–0.901; *p* = 0.002) lower than a Beninese woman who had never experienced IPV, controlling for relevant exploratory variables. Although IPV was statistically significantly associated with at least 8 ANC visits (Table 1), it is not a predictor of the likelihood of at least 8 visits in this model (OR, 0.865; 95% CI:0.646–1.159; *p* = 0.332).

**Table 2:** Logistic Regression Predicting Utilization of Antenatal Care in Benin (IDSB-V, 2017–2018)

Variable (reference group)	At least 4 visits				At least 8 visits			
	Odds Ratio	Stand. Error	Confidence Interval 95%	*p*-value	Odds Ratio	Stand. Error	Confidence Interval 95%	*p*-value
Ever Experienced IPV (never IPV)	0.753**	(0.0691)	0.628–0.901	0.002	0.865	(0.129)	0.646–1.159	0.332
Decision making (no decision making)^a^	1.280*	(0.132)	1.045–1.569	0.017	1.724**	(0.340)	1.170–2.540	0.0059
Age Group (15–24)								
25–34	1.157	(0.144)	0.906–1.477	0.241	1.882**	(0.397)	1.244–2.847	0.002
35–49	1.470*	(0.235)	1.074–2.013	0.016	2.194**	(0.572)	1.315–3.662	0.003
Lives in rural region (urban)	1.075	(0.125)	0.855–1.352	0.537	0.843	(0.142)	0.606–1.173	0.310
Respondent Education (no education)								
Primary	1.262	(0.186)	0.945–1.686	0.114	0.970	(0.187)	0.664–1.418	0.876
Secondary or Higher	3.806	(3.053)	0.788–18.40	0.096	1.313	(0.484)	0.636–2.710	0.461
Wealth Index (poorest)								
Poorer	1.587***	(0.196)	1.245–2.022	0.000	1.301	(0.395)	0.716–2.364	0.387
Middle	1.857***	(0.243)	1.437–2.401	0.000	1.934*	(0.586)	1.067–3.505	0.030
Rich	2.618***	(0.363)	1.994–3.438	0.000	2.618***	(0.749)	1.493–4.592	0.001
Richest	5.490***	(0.951)	3.907–7.714	0.000	5.780***	(1.732)	3.208–10.41	0.000
Respondent Employment								
Working (not working)	0.904	(0.143)	0.662–1.233	0.522	1.100	(0.298)	0.646–1.872	0.725
Paid for work (not paid for work)	1.451**	(0.190)	1.122–1.876	0.005	0.890	(0.229)	0.537–1.476	0.652
Number of ever children (one child)								
2–4 children	0.680**	(0.093)	0.519–0.891	0.005	0.573**	(0.122)	0.377–0.870	0.010
5 or more children	0.589**	(0.108)	0.411–0.846	0.004	0.542*	(0.153)	0.311–0.945	0.031
Religion (Muslim)								
Catholic	1.983***	(0.277)	1.507–2.610	0.000	1.651*	(0.393)	1.034–2.637	0.036
Christian	2.942***	(0.411)	2.236–3.870	0.000	2.838***	(0.626)	1.840–4.378	0.000
Traditional	1.717**	(0.287)	1.237–2.384	0.001	1.549	(0.564)	0.758–3.166	0.230
Other	2.990***	(0.559)	2.072–4.317	0.000	1.863*	(0.574)	1.017–3.412	0.040
No religion	1.687*	(0.350)	1.122–2.537	0.0121	2.840**	(1.129)	1.301–6.200	0.009
Husband/Partner Education (no education)								
Primary	1.165	(0.132)	0.932–1.456	0.179	1.440*	(0.253)	1.020–2.033	0.039
Secondary or Higher	1.738*	(0.411)	1.093–2.764	0.020	1.724*	(0.386)	1.110–2.678	0.015
Unknown	1.063	(0.244)	0.677–1.667	0.791	1.079	(0.366)	0.554–2.101	0.824
Husband employed (not employed)	1.066	(0.379)	0.531–2.144	0.856	1.015	(0.602)	0.317–3.252	0.980
Husband Drinks (does not drink)	0.890	(0.0938)	0.723–1.094	0.268	0.980	(0.180)	0.683–1.406	0.912
Wife-beating justified (not justified)	1.077	(0.103)	0.893–1.298	0.439	1.071	(0.164)	0.792–1.447	0.657
Constant	0.273**	(0.108)	0.125–0.594	0.001	0.0144***	(0.010)	0.00389–0.0534	0.000
Observations	3084				3084			

Standard error in parenthesis - *** *p* < 0.001, ** *p* < 0.01, * *p* < 0.05

Beninese women who made decisions regarding their own health care, large household purchases and visiting with relatives and family had 28% higher odds of using ANC-4 services (OR, 1.279; 95% CI: 1.045–1.569; *p* = 0.017) and 72% higher odds of using ANC-8 (OR, 1.72; 95% CI: 1.17–2.54; *p* = 0.006), than women whose husbands or others (i.e. kin relations) were making the decisions. Women 35–49 years of age were 1.5 times more likely to use ANC-4 (OR, 1.470; 95% CI: 1.074–2.013; *P* = 0.016) and 2.2 times more likely to use ANC-8 (OR, 2.194; 95% CI: 1.315–3.662; *p* = 0.003) than women aged 15–24 years.

All women from the first four wealth quintiles were more likely to use ANC-4 than the fifth (poorest) quintile. Notably, there was no significant difference between the poorer and poorest quintiles in accessing ANC-8. Women from the wealthiest quintile were more than 5.5 times likely to use this necessary service than the women in the poorest quintile (OR, 5.490; 95% CI: 3.907–7.714; *p* < 0.001 for ANC-4 and OR, 5.780; 95% CI: 3.208–10.41; *p* < 0.001 for ANC-8). Although working was not a significant factor for the women, being paid cash for working compared to not being paid cash (i.e. paid in-kind or not paid cash) increased the likelihood of using ANC-4 by 45% (OR, 1.451; 95% CI: 1.122–1.876, *p* = 0.005). Notably, being paid cash for work was not a significant factor for attending at least eight ANC visits.

Having two to four children or five or more children compared to one child reduced the likelihood of utilizing ANC-4 by 32% (OR, 0.680; 95% CI: 0.519–0.981, *p* = 0.005) and 41% (OR, 0.589; 95% CI: 0.411–0.846; *p* = 0.004), respectively. Similarly, more children decreased likelihood of accessing ANC-8 by 43% for women with two to four children (OR, 0.573; 95% CI: 0.377–0.870; *p* = 0.009) and 46% for women with five or more children compared to women with one child (OR, 0.542; 95% CI: 0.311–0.945; *P* = 0.03). All the religions were more likely to utilize ANC compared to the Muslim Beninese women for ANC-4. A similar pattern was seen for women accessing at least eight ANC visits with the exception of belonging to a Traditional religion, which was not a significant factor. Beninese women who indicated no religion were almost three times more likely than Muslim women to access at least eight visits (OR, 2.8; 95% CI: 1.301–6.200; *p* = 0.009).

Women whose husbands or partners had secondary or higher education were 1.7 times more likely to access ANC-4 compared to husbands or partners with no education (OR. 1.738; 95% CI; 1.093–2.764; *p* = 0.02). Women whose partners had primary as well as secondary/higher education were 40% and 72%, respectively, more likely to access at least 8-visits compared to partners with no education (OR 1.44; 95% CI: 1.020–2.033; *p* = 0.039 & OR, 1.724, 95% CI: 1.110–2.678; *p* = 0.015).

Partner or husband and women’s employment, women’s level of education as well as place of residence were not predictors of ANC-4 or ANC-8 utilization in Benin. Although alcohol drinking was associated with IPV (Table 1), it was not a predictor of ANC utilization when controlling for relevant variables (i.e. religion).

## Discussion

We undertook this study to further understand how IPV and other key factors are associated with underutilization of ANC, placing the fetus or newborn and mother at risk of complications during pregnancy, delivery and postnatally [23, 24]. We focused on Benin because of the underutilization of ANC and high prevalence of IPV [13, 16].

Our study showed that IPV had significant negative impacts on ANC-4 utilization for Beninese women in 2017–2018; experience of any form of violence in a lifetime led to 25% less odds of using ANC-4 services. When we analyzed the impact of IPV on ANC based on the WHO’s updated recommendation for at least eight ANC visits - contrary to what we saw with the basic 4-visit model - IPV was not a significant predictor of access to eight visits. To our knowledge, no study to date has looked at the impact of IPV on the updated WHO recommended eight visit model in LMICs. Studies using the basic four-visit ANC model and were conducted in LMICs revealed mixed findings. A meta-analysis of studies to date revealed experience of IPV had a 25% decreased odds of using adequate ANC with the four visit model [10]. Work in Pakistan, Mozambique, and Nigeria showed a significant association with reduced ANC utilization among women who experienced emotional, physical and or sexual IPV [5, 25, 26] while none was found in Tanzania and Rwanda [4, 27]. A possible explanation for an association between IPV and ANC-8, but no predictive relationship could be that other factors have not been considered and perhaps a path analysis would provide further insight into the relationship. In fact, path analysis could potentially explore another path where ANC could work preventatively for IPV. Furthermore, we have to consider the small number (10% of the sample) which accessed 8 visits; this makes it difficult to draw conclusions with a small representation of the sample. Future work should consider pooling DHS data from several years or across regions in LMICs to investigate the impact of IPV on accessing at least 8-visits.

Our study also revealed there was a higher chance of using ANC-4 and ANC-8 if the woman made decisions in the household about her own healthcare, visiting family, spending husband’s earnings, and large household purchases. Decision-making is widely used as an indicator for female autonomy and women’s empowerment in studies using DHS datasets [28]. A study of SSA showed a weak link between decision making and ANC utilization [29], yet, decision making power was seen to play a role in higher utilization in Bangladesh, suggesting better spousal cooperation on household and health issues [30]. A recent study in Nepal looked at the roles of both autonomy (i.e. decision making) and experience of IPV as predictors of ANC utilization suggesting no significant association and a need for further research to consider country specific contexts and social norms [31]. A 2019 study combined data from 63 countries in part to assess the role of women’s autonomy in maternal health utilization (accessing at least 8 visits) and found that the contribution to making decisions was associated with a 42% increased odds of receiving at least eight ANC visits [32].

Older age, 35–49 years, increased the odds of accessing both ANC-4 and ANC-8 in our study. A 2019 systematic review of determinants of antenatal care utilization in sub-Sharan Africa showed mixed findings in the region with regards to age groups [3]. In most of the studies, increasing maternal age increased utilization of at least one and four ANC visits, which is similar to our findings in Benin [3]. Interestingly, some studies did show that younger women had a higher chance of attending the first ANC visit in the first trimester of pregnancy, most likely due to childbearing inexperience and being newly married or an adolescent [3].

In our study, there was no significant difference among women who suffered violence, and those who had not in terms of their justification of wife beating. Similarly, this variable was not a significant predictor of utilization of ANC. A 2018 study in Benin looked at the perception of Beninese on IPV revealing one in six people considered that it was justified for a partner or husband to beat his wife/partner [14], reinforcing the important consideration of social norms when attempting to create policies and programs.

Cultural and religious beliefs play an integral role in the decision making process of seeking ANC [21]. Not surprisingly, religion had a significant influence on ANC utilization in our study, revealing that the predicted probability of Christian Beninese women using ANC-4 and ANC-8 was close to 3 times that of Muslim women. There is a need for religion to be integrated as part of the social determinants of health framework in policy decisions [33].

Poverty was a significant variable when controlling for all other variables. Compared to the poorest quintile, the wealthier quintiles had a higher chance of utilizing ANC-4 and ANC-8, particularly women in the richest quintile who had over 5 times the odds of ANC utilization. Dansou et al., (2017) found close to 40% of Beninese mothers received less than four ANC visits or never attended services, suggesting household wealth as one of the factors [16]. Concurrently, receiving cash for work in our study (instead of not being paid cash or not working) increased the chances of using ANC-4 [16]. These findings align with other studies that have suggested costs associated with ANC (i.e. transportation, diagnostics, supplements) dissuade the financially disadvantaged women from seeking care [34]. Interestingly, working or being paid cash for work was not a factor that predicted ANC-8 utilization, most likely because those who can access eight ANC visits most likely belong to the higher wealth quintiles.

Like other studies, rural residence was positively associated with IPV in Benin [35]. However, and similar to previous findings in Benin, rurality was not a predictor of ANC utilization [16]. Some scholars suggest the erosion of historical advantages enjoyed by urban areas over rural areas in Africa, such as livelihood opportunities and access to healthcare [36]. There was a strong association between partner drinking alcohol behavior and woman’s experience of IPV, as documented in other work [37]. Yet, drinking alcohol did not have a significant impact on the utilization of ANC. No study, to our knowledge, has shown alcohol as a significant predictor of ANC utilization. Pathway analysis in future work could determine a causal pathway between partner or husband alcohol consumption and ANC uptake and utilization.

The number of children was significant in predicting ANC utilization; our model revealed decreasing odds of ANC utilization as the number of children increased for both ANC models. Ali et al., (2010) suggest that women who have had children: (i) rely on their previous pregnancies therefore are less likely to think they need ANC, (ii) have responsibilities to care for their current children, and (iii) have had negative perceptions from previous ANC experiences; ultimately, dissuading them from utilizing the service [6].

Some studies have shown that male unemployment rate is associated with an increase in incidence of physical violence against women [38] or being at risk for male to female violence [39]. Interestingly, our study showed no difference in partner employment status of women who had experienced IPV compared to women who never experienced IPV. A possible explanation is that both groups of women, ever IPV and never IPV, in the study population had partners or husbands who are employed at the time of data collection, 99.1 and 98.7%, respectively. As this was not a main variable in our research question, we did not disaggregate the types of employment. Perhaps a further disaggregation of the types of jobs - high skilled stable employment versus low skill/manual labour and precarious/unstable employment - would provide further insight as this would potentially influence wealth status.

A strength of our study was the use of a large, nationally representative demographic and health survey (DHS) that has an established methodology. This is the first study, to our knowledge, which looked at the impact of IPV on ANC utilization using the recent 2017–2018 Benin DHS as well as the first that looked at its impact on both ANC-4 and ANC-8. Modeling revealed key micro, meso and macro level factors to consider for policy and decision makers in Benin. Programs and interventions can target and incorporate key factors at the relevant levels and have a better chance at improving ANC utilization. This study builds on the body of knowledge indicating IPV affects utilization of ANC which is relevant in light of the recent COVID-19 pandemic and emerging trends of increased IPV due to lock down and quarantine [40]. Our study has the following limitations to consider. It is cross-sectional and therefore limited to associations with no opportunity to formulate causal inferences. As a secondary database analysis, authors are limited to already existing definitions and available data collected through the established survey.

## Conclusion

There is evidence that Beninese women who experience emotional, physical or sexual violence were less likely to use ANC services. This work revealed key areas for maternal health policy makers and service providers in Benin, to appropriately plan the effective policies addressing underlying causes (i.e. alleviate poverty; equitable access to health services; cultural sensitivity) and necessary interventions (i.e. ANC education, IPV prevention, paid employment, alcohol drinking cessation). Violence and oppression of women leads to underutilization of ANC, increasing the odds of negative health outcomes for women and their newborns during pregnancy, at birth and postnatally.

## Supplementary Information

**Additional file 1.** STATA Syntax for data analysis.

## Data Availability

The datasets generated and/or analyzed during the current study are available through the publicly available Demographic and Health Survey (DHS) Program accessible at the following link: https://dhsprogram.com/.

## Abbreviations

ANC: Antenatal care
ANC-4: At least four ANC visit model
ANC-8: At least eight ANC visit model
BDHS: Benin Demographic and Health Survey
CI: Confidence interval
ICF: Inner City Fund
INSAE: Ministry of Planning and Development and the National Institute of Statistics and Economic Analysis
IPV: Intimate partner violence
LMICs: Low and middle income countries
OR: Odds ratio
SSA: Sub-Saharan Africa
UNAIDS: United Nations Programme on HIV/AIDS
UNICEF: United Nations International Children’s Emergency Fund
UFPA: United Nations Fund for Population Activities
WHO: World health organization
