# Supplementary material for: Exploring antenatal care utilization and intimate partner violence in Benin - are lives at stake?
Source: BMC Public Health. 2021 Apr 30;21:830. doi: 10.1186/s12889-021-10884-9 (PMC8085473; doi:10.1186/s12889-021-10884-9)
Supplement: Supplementary file 1 — Additional file 1. STATA Syntax for data analysis. [file 12889_2021_10884_MOESM1_ESM.pdf]

```
*********************************************
*Research Question Using basic 4 visit model*
*********************************************

*What is the effect of intimate partner violence on utilization of antinatal care services in Benin?*

*********************************
*Determining sample*
*********************************
*tab the number of women who completed the question on antenatal care utilization with women who completed the domestic violence module*
codebook m14_1
tab m14_1, m

drop if m14_1==.
tab m14_1, m

*women who answered ANC questions and completed domestic violence module = 3397*

*********************************
*   RECODING VARIABLES  *
*********************************

********************************************************************************
*DEPENDENT VARIABLES: ANTENATAL CARE UTILIZATION
********************************************************************************
*Number of antenatal visits during the pregnancy. Women who did not see anyone for antenatal care during the pregnancy are coded 0
*The focused ANC (FANC) model, also known as the basic ANC model, includes four ANC visits occurring between 8 and 12 weeks of gestation, between 24 and 26 weeks, at 32 weeks, and between 36 and 38 weeks. 2002 recommendation by WHO. Guidance on each visit includes specific evidence-based interventions for healthy pregnant women (called “goal-oriented”), with appropriate referral of high-risk women and those who develop pregnancy complications. The number of visits in this model is considerably fewer than in ANC models used in HICs. https://extranet.who.int/rhl/topics/improving-health-system-performance/who-recommendation-antenatal-care-contact-schedules


*Antenatal Care Service (ANC) Utilization Focused ANC & Basic Model with at least 8 ANC visits - Dependent #1*
```

```
********************

*experienced any emotional violence*
codebook d104
tab d104, m
recode d104 (1=1 "YES")(2 = 0 "No") (99=.) (.=.), gen(dumEV_IVMISS)
tab d104 dumEV_IVMISS, m

*Physical Violence*
*******************
*ever been pushed, shook or had something thrown by husband/partner*
codebook d105a
tab d105a, m
recode d105a (0=0 "NO")(1/3 = 1 "YES") (99=.) (.=.), gen(
dumPV_IVMISS_a)
tab d105a dumPV_IVMISS_a, m

*ever been slapped by husband/partner*
codebook d105b
tab d105b, m
recode d105b (0=0 "NO")(1/3 = 1 "YES") (99=.) (.=.), gen(
dumPV_IVMISS_b)
tab d105b dumPV_IVMISS_b, m

*ever been punched with fist or hit by something harmful by
husband/partner*
codebook d105c
tab d105c, m
recode d105c (0=0 "NO")(1/3 = 1 "YES") (99=.) (.=.), gen(
dumPV_IVMISS_c)
tab d105c dumPV_IVMISS_c, m

*ever been kicked or dragged by husband/partner*
codebook d105d
tab d105d, m
recode d105d (0=0 "NO")(1/3 = 1 "YES") (99=.) (.=.), gen(
dumPV_IVMISS_d)
tab d105d dumPV_IVMISS_d, m

*ever been strangled or burnt by husband/partner*
codebook d105e
tab d105e, m
recode d105e (0=0 "NO")(1/3 = 1 "YES") (99=.) (.=.), gen(
dumPV_IVMISSV_e)
tab d105e dumPV_IVMISSV_e, m

*ever been threatened with knife/gun or other weapon by
husband/partner*
```

```
*ever been forced into other unwanted sexual acts by
husband/partner*
codebook d105i
tab d105i, m
recode d105i (0=0 "NO")(1/3 = 1 "YES") (99=.) (.=.), gen(
dumSV_IVMISS_i)
tab d105i dumSV_IVMISS_i, m

*ever been physically forced to perform sexual acts respondent
didn't want to*
codebook d105k
tab d105k, m
recode d105k (0=0 "NO")(1/3 = 1 "YES") (99=.) (.=.), gen(
dumSV_IVMISS_k)
tab d105k dumSV_IVMISS_k, m

*INDEPENDENT VARIABLE DUMMY FOR IPV*
gen sumdumIV = dumEV_IVMISS+dumPV_IVMISS_a+dumPV_IVMISS_b+
dumPV_IVMISS_c+dumPV_IVMISS_d+dumPV_IVMISSV_e+dumPV_IVMISS_f+
dumPV_IVMISS_j+dumSV_IVMISS_h+dumSV_IVMISS_i+dumSV_IVMISS_k
tab sumdumIV, m

recode sumdumIV (0=0 "Never IPV") (1/11=1 "Ever IPV") (.=.), gen (
dumeverIPV)
tab dumeverIPV, m

recode sumdumIV (0=1 "Never IPV") (1/11=0 "Ever IPV") (.=.), gen (
dumNeverIPV)
tab dumNeverIPV, m

tab dumeverIPV dumuseBasicANC_4, m


***********************************
*EXPLORATORY INDEPENDENT VARIABLES*
***********************************

*Generate Dummy for Decision Making/Empowerment/Autonomy**

*Decision-making questions*
*********************

*person who usually decides on respondent's health care*
codebook v743a
tab v743a, m
recode v743a (1 2 =1 "some decisions") (4 5 6=0 "no decisions") (99
=.) (.=.), gen (dumauthealth)
tab v743a dumauthealth, m
```

```
tab v743f dumauthusearn, m

*Decision making DUMMY*
*Rationale for using the four variables for autonomy based on *
*Osamor, P. E., & Grady, C. (2016). Women’s autonomy in health
care decision-making in developing countries:
*A synthesis of the literature. International Journal of Women’s
Health, 8, 191–202. https://doi.org/10.2147/IJWH.S105483*
gen IFScale = dumauthealth + dumautlgpuch + dumautvisitfam +
dumauthusearn
tab IFScale, m

recode IFScale (0=1 "no decisions") (1/11=0 "some decisions")
(.=.), gen (dumNODecisions)
tab IFScale dumNODecisions, m

recode IFScale (0=0 "no decisions") (1/11=1 "some decisions")
(.=.), gen (dumDecisions)
tab IFScale dumDecisions, m

tab dumDecisions

*OTHER EXPLORATORY VARIABLES

*Age of Respondent in categories*
recode v012 (15/24= 1 "15-24") (25/49=0 "Other")(99=.) (.=.), gen (
dumAge_15_24)
tab v012 dumAge_15_24, m

recode v012 (15/24 35/49= 0 "Othr") (25/34=1 "25/34")(99=.) (.=.),
gen (dumAge_25_34)
tab v012 dumAge_25_34, m

recode v012 (35/49= 1 "35-49") (15/34=0 "Other")(99=.) (.=.), gen (
dumAge_35_49)
tab v012 dumAge_35_49, m

*Residence*
codebook v102
tab v102, m

recode v102 (2=1 "Rural") (1=0 "Urban") (99=.) (.=.), gen (dumrural)
tab v102 dumrural, m

recode v102 (2=0 "Rural") (1=1 "Urban") (99=.) (.=.), gen (dumurban)
tab v102 dumurban, m

tab dumrural
```

```
*Wealth Index*
codebook v190
tab v190, m

recode v190 (1=1 "poorest") (2/5=0 "other")(99=.) (.=.), gen (dumpoorest)
tab v190 dumpoorest, m

recode v190 (2=1 "poorer") (1 3 4 5 =0 "other")(99=.) (.=.), gen (dumpoorer)
tab v190 dumpoorer, m

recode v190 (3=1 "middle") (1 2 4 5 =0 "other")(99=.) (.=.), gen (dummiddle)
tab v190 dummiddle, m

recode v190 (4=1 "rich") (1 2 3 5 =0 "other")(99=.) (.=.), gen (dumrich)
tab v190 dumrich, m

recode v190 (5=1 "richest") (1 2 3 4 =0 "other")(99=.) (.=.), gen (dumrichest)
tab v190 dumrichest, m

*Working Woman*
codebook v714
tab v714, m
recode v714 (0=0 "Not Working") (1=1 "Working") (99=.) (.=.), gen (dumworkingwoman)
tab v714 dumworkingwoman, m

recode v714 (0=1 "Not Working") (1=0 "Working") (99=.) (.=.), gen (dumNOTworkingwoman)
tab v714 dumNOTworkingwoman, m

*Type of earnings
codebook v741
tab v741

recode v741 (0 3 .=1 "Not Paid Cash") (1/2 =0 "Paid") (99=.), gen (dumNOTPaidorWork)
tab v741 dumNOTPaidorWork, m

recode v741 (0 3 .=0 "Not Paid Cash") (1/2=1 "Paid ") (99=.), gen (dumPaid)
tab v741 dumPaid, m

*recode v741 (.=1 "Not Working") (1/3=0 "Working") (99=.), gen
```

```
tab v729 dumhusSECedPLUS, m

recode v729 (8=1 "don't know'") (0/5 =0 "other")(99=.) (.=.), gen (
dumhusedUnknown_B)
tab v729 dumhusedUnknown_B, m


*Employment_Husband/Partner*
codebook v705
tab v705, m

recode v705 (0=0 "Not Employed") (1/98 = 1 "Employed")(99=.) (.=.),
 gen (dumhusemp)
tab v705 dumhusemp, m

recode v705 (0=1 "Not Employed") (1/98 = 0 "Employed")(99=.) (.=.),
 gen (dumhusNOTemp)
tab v705 dumhusNOTemp, m

*husband/partner drinks alcohol*
codebook d113
tab d113, m

recode d113 (0=0 "No Drink") (1=1 "Yes Drink") (99=.) (.=.), gen (
dumdrink)
tab d113 dumdrink, m

recode d113 (0=1 "No Drink") (1=0 "Yes Drink") (99=.) (.=.), gen (
dumNOdrink)
tab d113 dumNOdrink, m

*Number of Children ever born
codebook v201
tab v201, m

recode v201 (1=1 "one child") (2/16=0 "Other") (99=.) (.=.), gen (
dumOneKid)
tab v201 dumOneKid, m

recode v201 (2/4=1 "2-4 Children") (1 5/16=0 "Other") (99=.) (.=.),
 gen (dum2to4Kids)
tab v201 dum2to4Kids, m

recode v201 (5/16=1 "5+ Children") (1/4=0 "Other") (99=.) (.=.),
gen (dum5plusKids)
tab v201 dum5plusKids, m
```

```
recode v130 (7 9 =1 "Other Religions") (1/6 8 10=0 "Other") (99=.) (.=.), gen (dumOther_Relig)
tab v130 dumOther_Relig

recode v130 (10 =1 "No Religion") (1/9=0 "Other") (99=.) (.=.), gen (dumNORelig)
tab v130 dumNORelig

*Beating Justified

*beating justified if wife goes out without telling husband
codebook v744a
tab v744a
recode v744a (1=1 "yes") (0 8=0 "no or I don't know") (99=.) (.=.), gen (dumBeatOut)
tab v744a dumBeatOut, m

*beating justified if wife neglects the children
codebook v744b
recode v744b (1=1 "yes") (0 8=0 "no or I don't know'") (99=.) (.=.), gen (dumBeatNegChild)
tab v744b dumBeatNegChild, m

*beating justified if wife argues with husband/partner
codebook v744c
recode v744c (1=1 "yes") (0 8=0 "no or I don't know'") (99=.) (.=.), gen (dumBeatArgueHusb)
tab v744c dumBeatArgueHusb, m

*beating justified if wife refuses to have sex with husband/partner
codebook v744d
recode v744d (1=1 "yes") (0 8=0 "no or I don't know'") (99=.) (.=.), gen (dumBeaNoSex)
tab v744d dumBeaNoSex, m

*beating justified if wife burns the food
codebook v744e
recode v744e (1=1 "yes") (0 8=0 "no or I don't know'") (99=.) (.=.), gen (dumBeatBurnFood)
tab v744e dumBeatBurnFood, m

gen IFScaleBeat = dumBeatOut + dumBeatNegChild + dumBeatArgueHusb + dumBeaNoSex +dumBeatBurnFood
tab IFScaleBeat, m

recode IFScaleBeat (1/5=1 "Justified") (0=0 "Not Justified")(99=.) (.=.), gen (dumBeatJustified)
tab IFScaleBeat dumBeatJustified
```

```
ci means dumuseBasicANC_4 dumeverIPV dumDecisions dumNODecisions
dumAge_15_24 dumAge_25_34 dumAge_35_49 dumrural dumurban
dumeNOeduc_B dumPRIMed_B dumSECedPlus ///
dumpoorest dumpoorer dummiddle dumrich dumrichest dumworkingwoman
dumNOTworkingwoman dumNOTPaidorWork dumPaid dumhusNOeduc_B
dumhusPRIMed_B dumhusSECedPLUS dumhusedUnknown_B dumhusemp
dumhusNOTemp dumdrink dumNOdrink dumOneKid dum2to4Kids dum5plusKids
 dumTradRelig dumIslam dumCatholic dumChristian dumOther_Relig
dumNORelig dumBeatJustified if dumeverIPV==1, level(95)


*******************************
*  DEALING WITH MISSING DATA  *
*******************************

* drop missing *  this order
drop if dumuseBasicANC_4==.
drop if dumeverIPV==.
drop if dumDecisions==.

ci means dumuseBasicANC_4 dumeverIPV dumDecisions dumNODecisions
dumAge_15_24 dumAge_25_34 dumAge_35_49 dumrural dumurban
dumeNOeduc_B dumPRIMed_B dumSECedPlus ///
dumpoorest dumpoorer dummiddle dumrich dumrichest dumworkingwoman
dumNOTworkingwoman dumNOTPaidorWork dumPaid dumhusNOeduc_B
dumhusPRIMed_B dumhusSECedPLUS dumhusedUnknown_B dumhusemp
dumhusNOTemp dumdrink dumNOdrink dumOneKid dum2to4Kids dum5plusKids
 dumTradRelig dumIslam dumCatholic dumChristian dumOther_Relig
dumNORelig dumBeatJustified if dumeverIPV==0, level(95)


ci means dumuseBasicANC_4 dumeverIPV dumDecisions dumNODecisions
dumAge_15_24 dumAge_25_34 dumAge_35_49 dumrural dumurban
dumeNOeduc_B dumPRIMed_B dumSECedPlus ///
dumpoorest dumpoorer dummiddle dumrich dumrichest dumworkingwoman
dumNOTworkingwoman dumNOTPaidorWork dumPaid dumhusNOeduc_B
dumhusPRIMed_B dumhusSECedPLUS dumhusedUnknown_B dumhusemp
dumhusNOTemp dumdrink dumNOdrink dumOneKid dum2to4Kids dum5plusKids
 dumTradRelig dumIslam dumCatholic dumChristian dumOther_Relig
dumNORelig dumBeatJustified if dumeverIPV==1, level(95)

*SAMPLE SIZE = 3084 cases

*********************************
*      Multicollinearity        *
*********************************
*checking out of interest where IPV is DP*

```

```
*v005 for individual records (IR) women's weights - have to take in to consideration the sample design
**PSU - enumeration area or sample cluster; STRATA  - read Appendix A.
*These two numbers are different, simply because the number of children born in the past five years is not statistically independent of the weight variable. The difference is very small, but some difference is virtually inevitable. The weighted number of cases is not a "population size". Proportions and means calculated with weights will be better estimates of population characteristics than the UNweighted proportions and means would be, but you cannot interpret a weighted frequency as a "population size. https://userforum.dhsprogram.com/index.php?t=msg&th=5560&start=0&S=Google"
gen wgt=v005/1000000
tab v005 [iweight=wgt]
svyset [pw=wgt], psu(v021) strata(v022)
tab v007
tab v007[iweight=v005/1000000]

* After you have determined your models and have everything set,
* the final step is to re-run your analyses using bootstrap weights
* (you can do this all along but it's very slow to run)
* You may need to readjust your models if the results are quite different

***** Redo all analyses using one of the svy commands:
*svy: tabulate
*svy: mean
*svy: regress
*svy: logit


* for means split by main IV to figure our the weighted means:
svy, subpop (dumeverIPV): mean dumuseBasicANC_4
svy, subpop (dumNeverIPV): mean dumuseBasicANC_4

svy, subpop (dumeverIPV): mean dumDecisions
svy, subpop (dumNeverIPV): mean dumDecisions

svy, subpop (dumeverIPV): mean dumAge_15_24
svy, subpop (dumNeverIPV): mean dumAge_15_24
svy, subpop (dumeverIPV): mean dumAge_25_34
svy, subpop (dumNeverIPV): mean dumAge_25_34
svy, subpop (dumeverIPV): mean dumAge_35_49
svy, subpop (dumNeverIPV): mean dumAge_35_49
```

```
svy, subpop (dumNeverIPV): mean dumrichest

svy, subpop (dumeverIPV): mean dumworkingwoman
svy, subpop (dumNeverIPV): mean dumworkingwoman

svy, subpop (dumeverIPV): mean dumhusNOeduc_B
svy, subpop (dumNeverIPV): mean dumhusNOeduc_B
svy, subpop (dumeverIPV): mean dumhusPRIMed_B
svy, subpop (dumNeverIPV): mean dumhusPRIMed_B
svy, subpop (dumeverIPV): mean dumhusSECedPLUS
svy, subpop (dumNeverIPV): mean dumhusSECedPLUS
svy, subpop (dumeverIPV): mean dumhusedUnknown_B
svy, subpop (dumNeverIPV): mean dumhusedUnknown_B

svy, subpop (dumeverIPV): mean dumhusemp
svy, subpop (dumNeverIPV): mean dumhusemp

svy, subpop (dumeverIPV): mean dumdrink
svy, subpop (dumNeverIPV): mean dumdrink

svy, subpop (dumeverIPV): mean dumOneKid
svy, subpop (dumNeverIPV): mean dumOneKid
svy, subpop (dumeverIPV): mean dum2to4Kids
svy, subpop (dumNeverIPV): mean dum2to4Kids
svy, subpop (dumeverIPV): mean dum5plusKids
svy, subpop (dumNeverIPV): mean dum5plusKids

svy, subpop (dumeverIPV): mean dumNOTPaidorWork
svy, subpop (dumNeverIPV): mean dumNOTPaidorWork
svy, subpop (dumeverIPV): mean dumPaid
svy, subpop (dumNeverIPV): mean dumPaid

svy, subpop (dumeverIPV) : mean dumTradRelig
svy, subpop (dumNeverIPV): mean dumTradRelig
svy, subpop (dumeverIPV) : mean dumIslam
svy, subpop (dumNeverIPV): mean dumIslam
svy, subpop (dumeverIPV) : mean dumCatholic
svy, subpop (dumNeverIPV): mean dumCatholic
svy, subpop (dumeverIPV) : mean dumChristian
svy, subpop (dumNeverIPV): mean dumChristian
svy, subpop (dumeverIPV) : mean dumOther_Relig
svy, subpop (dumNeverIPV): mean dumOther_Relig
svy, subpop (dumeverIPV) : mean dumNORelig
svy, subpop (dumNeverIPV): mean dumNORelig

svy, subpop (dumeverIPV) : mean dumBeatJustified
svy, subpop (dumNeverIPV): mean dumBeatJustified
```

```
svy: logit dumSECedPlus dumeverIPV

svy: logit dumpoorest dumeverIPV
svy: logit dumpoorer dumeverIPV
svy: logit dummiddle dumeverIPV
svy: logit dumrich dumeverIPV
svy: logit dumrichest dumeverIPV
svy: logit dumworkingwoman dumeverIPV

svy: logit dumhusNOeduc_B dumeverIPV
svy: logit dumhusPRIMed_B dumeverIPV
svy: logit dumhusSECedPLUS dumeverIPV
svy: logit dumhusedUnknown_B dumeverIPV

svy: logit dumhusemp dumeverIPV

svy: logit dumdrink dumeverIPV

svy: logit dumPaid dumeverIPV
svy: logit dumNOTPaidorWork dumeverIPV

svy: logit dumOneKid  dumeverIPV
svy: logit dum2to4Kids dumeverIPV
svy: logit dum5plusKids dumeverIPV

svy: logit dumTradRelig dumeverIPV
svy: logit dumIslam  dumeverIPV
svy: logit dumCatholic  dumeverIPV
svy: logit dumChristian dumeverIPV
svy: logit dumOther_Relig  dumeverIPV
svy: logit dumNORelig dumeverIPV

svy: logit dumBeatJustified dumeverIPV


* REGRESSION ANALYSIS WITH WEIGHTED DATA

svy: logistic dumuseBasicANC_4 dumeverIPV dumDecisions dumAge_25_34
 dumAge_35_49 dumrural dumPRIMed_B dumSECedPlus dumpoorer dummiddle
 dumrich dumrichest dumworkingwoman dumPaid dumhusPRIMed_B
dumhusSECedPLUS dumhusedUnknown_B dumhusemp dumdrink dum2to4Kids
dum5plusKids  dumCatholic dumChristian dumTradRelig dumOther_Relig
dumNORelig dumBeatJustified

outreg2 using TakeJan_8.xls,  alpha (0.001, 0.01, 0.05) symbol(***,
 **, *) append sideway stats(coef se ci pval) eform
```

```
drop if m14_1==.
tab m14_1, m

*women who answered ANC questions and completed domestic violence module = 3397*

*********************************
*   RECODING VARIABLES  *
*********************************

********************************************************************************
*DEPENDENT VARIABLES: ANTENATAL CARE UTILIZATION
*Antenatal Care Service (ANC) Utilization Focused ANC & Basic Model with at least 8 ANC visits - Dependent #1*
codebook m14_1
tab m14_1, m

*AT LEAST 8 VISITS*
recode m14_1 (0/7 = 0 "No ANC Utilization") (8/20 = 1 "ANC Utilization") (98 99=.) (.=.), gen (dumuseBasicANC_8)
tab m14_1 dumuseBasicANC_8, m

recode m14_1 (0/7 = 1 "No ANC Utilization") (8/20 = 0 "ANC Utilization") (98 99=.) (.=.), gen (dumuseNOBasicANC_8)
tab m14_1 dumuseNOBasicANC_8, m

tab dumuseBasicANC_8
********************************************************************************
*INDEPENDENT VARIABLE: INTIMATE PARTNER VIOLENCE & AUTONOMY*
********************************************************************************

*Generate Dummy for Intimate Partner Violence *

*Emotional Violence*
********************

*experienced any emotional violence*
codebook d104
tab d104, m
recode d104 (1=1 "YES")(2 = 0 "No") (99=.) (.=.), gen(dumEV_IVMISS)
tab d104 dumEV_IVMISS, m

*Physical Violence*
*******************
*ever been pushed, shook or had something thrown by husband/partner*
```

```
codebook d105d
tab d105d, m
recode d105d (0=0 "NO")(1/3 = 1 "YES") (99=.) (.=.), gen(
dumPV_IVMISS_d)
tab d105d dumPV_IVMISS_d, m

*ever been strangled or burnt by husband/partner*
codebook d105e
tab d105e, m
recode d105e (0=0 "NO")(1/3 = 1 "YES") (99=.) (.=.), gen(
dumPV_IVMISSV_e)
tab d105e dumPV_IVMISSV_e, m

*ever been threatened with knife/gun or other weapon by
husband/partner*
codebook d105f
tab d105f, m
recode d105f (0=0 "NO")(1/3 = 1 "YES") (99=.) (.=.), gen(
dumPV_IVMISS_f)
tab d105f dumPV_IVMISS_f, m

*ever had arm twisted or hair pulled by husband/partner*
codebook d105j
tab d105j, m
recode d105j (0=0 "NO")(1/3 = 1 "YES")(99=.) (.=.), gen(
dumPV_IVMISS_j)
tab d105j dumPV_IVMISS_j, m


*Sexual Violence*
*****************

*ever been physically forced into unwanted sex by husband/partner*
codebook d105h
tab d105h, m
recode d105h (0=0 "NO")(1/3 = 1 "YES") (99=.) (.=.), gen(
dumSV_IVMISS_h)
tab d105h dumSV_IVMISS_h, m

*ever been forced into other unwanted sexual acts by
husband/partner*
codebook d105i
tab d105i, m
recode d105i (0=0 "NO")(1/3 = 1 "YES") (99=.) (.=.), gen(
dumSV_IVMISS_i)
tab d105i dumSV_IVMISS_i, m

*ever been physically forced to perform sexual acts respondent
```

```
***********************************

*Generate Dummy for Decision Making/Empowerment/Autonomy**

*Decision-making questions*
*********************

*person who usually decides on respondent's health care*
codebook v743a
tab v743a, m
recode v743a (1 2 =1 "some decisions") (4 5 6=0 "no decisions") (99 =.) (.=.), gen (dumauthealth)
tab v743a dumauthealth, m

*person who usually decides on large household purchases*
codebook v743b
tab v743b
recode v743b (1 2=1 "some decisions") (4 5 6=0 "no decisions") (99 =.) (.=.), gen (dumautlgpuch)
tab v743b dumautlgpuch, m

*person who usually decides on visits to family or relatives*
codebook v743d
tab v743d
recode v743d (1 2=1 "some decisions") (4 5 6=0 "no decisions") (99 =.) (.=.), gen (dumautvisitfam)
tab v743d dumautvisitfam, m

*REVISIT*****person who usually decides what to do with money husband earns*
codebook v743f
tab v743f
recode v743f (1 2=1 "some decisions") (4 6 7=0 "no decisions") (99 =.) (.=.), gen (dumauthusearn)
tab v743f dumauthusearn, m

*Decision making DUMMY*
*Rationale for using the four variables for autonomy based on *
*Osamor, P. E., & Grady, C. (2016). Women's autonomy in health care decision-making in developing countries:
*A synthesis of the literature. International Journal of Women's Health, 8, 191–202. https://doi.org/10.2147/IJWH.S105483*
gen IFScale = dumauthealth + dumautlgpuch + dumautvisitfam + dumauthusearn
tab IFScale, m

recode IFScale (0=1 "no decisions") (1/11=0 "some decisions") (.=.), gen (dumNODecisions)
```

```
tab v012 dumAge_25_34, m

recode v012 (35/49= 1 "35-49") (15/34=0 "Other")(99=.) (.=.), gen (
dumAge_35_49)
tab v012 dumAge_35_49, m

*Residence*
codebook v102
tab v102, m

recode v102 (2=1 "Rural") (1=0 "Urban") (99=.) (.=.), gen (dumrural)
tab v102 dumrural, m

recode v102 (2=0 "Rural") (1=1 "Urban") (99=.) (.=.), gen (dumurban)
tab v102 dumurban, m

tab dumrural

*Education
codebook v149
tab v149, m

recode v149 (0/1=1 "no education") (2/5=0 "other") (99=.) (.=.),
gen (dumeNOeduc_B)
tab v149 dumeNOeduc_B, m

recode v149 (2/3=1 "primary education") (0/1 4/5=0 "other") (99=.)
(.=.), gen (dumPRIMed_B)
tab v149 dumPRIMed_B, m

recode v149 (4/5=1 "secondary education or higher") (0 1 2 3 =0
"other") (99=.) (.=.), gen (dumSECedPlus)
tab v149 dumSECedPlus, m


*Wealth Index*
codebook v190
tab v190, m

recode v190 (1=1 "poorest") (2/5=0 "other")(99=.) (.=.), gen (
dumpoorest)
tab v190 dumpoorest, m

recode v190 (2=1 "poorer") (1 3 4 5 =0 "other")(99=.) (.=.), gen (
dumpoorer)
tab v190 dumpoorer, m

recode v190 (3=1 "middle") (1 2 4 5 =0 "other")(99=.) (.=.), gen (
```

```
recode v714 (0=1 "Not Working") (1=0 "Working") (99=.) (.=.), gen (
dumNOTworkingwoman)
tab v714 dumNOTworkingwoman, m

*Type of earnings
codebook v741
tab v741

recode v741 (0 3 .=1 "Not Paid Cash") (1/2 =0 "Paid") (99=.), gen (
dumNOTPaidorWork)
tab v741 dumNOTPaidorWork, m

recode v741 (0 3 .=0 "Not Paid Cash") (1/2=1 "Paid ") (99=.), gen (
dumPaid)
tab v741 dumPaid, m

*recode v741 (.=1 "Not Working") (1/3=0 "Working") (99=.), gen
(dumNotPAIDWorking)
*tab v741 dumNotPAIDWorking, m

*tab v741 v714, m
*The missing 3525 from the Not paid question are NOT Working* so I
am going to include in Not working on their own.

* Husband/Partner Education
codebook v729
tab v729, m

recode v729 (0/1=1 "no education") (2/5 8=0 "other") (99=.) (.=.),
gen (dumhusNOeduc_B)
tab v729 dumhusNOeduc_B, m

recode v729 (2/3=1 "primary education") (0 1 4/5 8=0 "other") (99
=.) (.=.), gen (dumhusPRIMed_B)
tab v729 dumhusPRIMed_B, m

recode v729 (4/5=1 "secondary education") (0 1 2 3 8=0 "other") (99
=.) (.=.), gen (dumhusSECedPLUS)
tab v729 dumhusSECedPLUS, m

recode v729 (8=1 "don't know'") (0/5 =0 "other")(99=.) (.=.), gen (
dumhusedUnknown_B)
tab v729 dumhusedUnknown_B, m


*Employment_Husband/Partner*
codebook v705
tab v705, m
```

```
tab v705 dumhusemp_b, m

recode v705 (0=1 "Not Employed") (1/98 = 0 "Other")(99=.) (.=.), gen (dumhusNOTemp_b)
tab v705 dumhusNOTemp_b, m

recode v705 (98=1 "Unknown") (1/96 = 0 "other")(99=.) (.=.), gen ( dumhusempunknown_b)
tab v705 dumhusempunknown_b, m



*husband/partner drinks alcohol*
codebook d113
tab d113, m

recode d113 (0=0 "No Drink") (1=1 "Yes Drink") (99=.) (.=.), gen ( dumdrink)
tab d113 dumdrink, m

recode d113 (0=1 "No Drink") (1=0 "Yes Drink") (99=.) (.=.), gen ( dumNOdrink)
tab d113 dumNOdrink, m

*Number of Children ever born
codebook v201
tab v201, m

recode v201 (1=1 "one child") (2/16=0 "Other") (99=.) (.=.), gen ( dumOneKid)
tab v201 dumOneKid, m

recode v201 (2/4=1 "2-4 Children") (1 5/16=0 "Other") (99=.) (.=.), gen (dum2to4Kids)
tab v201 dum2to4Kids, m

recode v201 (5/16=1 "5+ Children") (1/4=0 "Other") (99=.) (.=.), gen (dum5plusKids)
tab v201 dum5plusKids, m


*Religion
codebook v130
tab v130
recode v130 (1/2=1 "traditional") (3/10=0 "Other") (99=.) (.=.), gen (dumTradRelig)
tab v130 dumTradRelig

```

```
tab v130 dumNORelig

*Beating Justified

*beating justified if wife goes out without telling husband
codebook v744a
tab v744a
recode v744a (1=1 "yes") (0 8=0 "no or I don't know") (99=.) (.=.), gen (dumBeatOut)
tab v744a dumBeatOut, m

*beating justified if wife neglects the children
codebook v744b
recode v744b (1=1 "yes") (0 8=0 "no or I don't know'") (99=.) (.=.), gen (dumBeatNegChild)
tab v744b dumBeatNegChild, m

*beating justified if wife argues with husband/partner
codebook v744c
recode v744c (1=1 "yes") (0 8=0 "no or I don't know'") (99=.) (.=.), gen (dumBeatArgueHusb)
tab v744c dumBeatArgueHusb, m

*beating justified if wife refuses to have sex with husband/partner
codebook v744d
recode v744d (1=1 "yes") (0 8=0 "no or I don't know'") (99=.) (.=.), gen (dumBeaNoSex)
tab v744d dumBeaNoSex, m

*beating justified if wife burns the food
codebook v744e
recode v744e (1=1 "yes") (0 8=0 "no or I don't know'") (99=.) (.=.), gen (dumBeatBurnFood)
tab v744e dumBeatBurnFood, m

gen IFScaleBeat = dumBeatOut + dumBeatNegChild + dumBeatArgueHusb + dumBeaNoSex +dumBeatBurnFood
tab IFScaleBeat, m

recode IFScaleBeat (1/5=1 "Justified") (0=0 "Not Justified")(99=.) (.=.), gen (dumBeatJustified)
tab IFScaleBeat dumBeatJustified

***********************************
*  Calculate Confidence Intervals *
***********************************

ci means dumuseBasicANC_8 dumeverIPV dumDecisions dumNODecisions
```

```
dumhusPRIMed_B dumhusSECedPLUS dumhusedUnknown_B dumhusemp
dumhusNOTemp dumdrink dumNOdrink dumOneKid dum2to4Kids dum5plusKids
 dumTradRelig dumIslam dumCatholic dumChristian dumOther_Relig
dumNORelig dumBeatJustified if dumeverIPV==1, level(95)

*******************************
*  DEALING WITH MISSING DATA  *
*******************************

* drop missing *  this order
drop if dumuseBasicANC_8==.
drop if dumeverIPV==.
drop if dumDecisions==.

ci means dumuseBasicANC_8 dumeverIPV dumDecisions dumNODecisions
dumAge_15_24 dumAge_25_34 dumAge_35_49 dumrural dumurban
dumeNOeduc_B dumPRIMed_B dumSECedPlus ///
dumpoorest dumpoorer dummiddle dumrich dumrichest dumworkingwoman
dumNOTworkingwoman dumNOTPaidorWork dumPaid dumhusNOeduc_B
dumhusPRIMed_B dumhusSECedPLUS dumhusedUnknown_B dumhusemp
dumhusNOTemp dumdrink dumNOdrink dumOneKid dum2to4Kids dum5plusKids
 dumTradRelig dumIslam dumCatholic dumChristian dumOther_Relig
dumNORelig dumBeatJustified if dumeverIPV==0, level(95)

ci means dumuseBasicANC_8 dumeverIPV dumDecisions dumNODecisions
dumAge_15_24 dumAge_25_34 dumAge_35_49 dumrural dumurban
dumeNOeduc_B dumPRIMed_B dumSECedPlus ///
dumpoorest dumpoorer dummiddle dumrich dumrichest dumworkingwoman
dumNOTworkingwoman dumNOTPaidorWork dumPaid dumhusNOeduc_B
dumhusPRIMed_B dumhusSECedPLUS dumhusedUnknown_B dumhusemp
dumhusNOTemp dumdrink dumNOdrink dumOneKid dum2to4Kids dum5plusKids
 dumTradRelig dumIslam dumCatholic dumChristian dumOther_Relig
dumNORelig dumBeatJustified if dumeverIPV==1, level(95)

*SAMPLE SIZE = 3084 cases

*********************************
*       Multicollinearity       *
*********************************

reg dumuseBasicANC_8  dumeverIPV dumDecisions dumAge_15_24 dumrural
 dumeNOeduc dumpoorest dumworkingwoman dumPaid dumhusNOeduc
dumhusemp dumdrink dumOneKid dumIslam dumBeatJustified
vif

* VIF<3 is great
* VIF 10+ is terrible
* 1/VIF (Tolerance STatistics)<0.10 is bad
```

```
characteristics than the UNweighted proportions and means would
be, but you cannot interpret a weighted frequency as a "population
size.
https://userforum.dhsprogram.com/index.php?t=msg&th=5560&start=0&S=G
oogle"
gen wgt=v005/1000000
tab v005 [iweight=wgt]
svyset [pw=wgt], psu(v021) strata(v022)
tab v007
tab v007[iweight=v005/1000000]

* After you have determined your models and have everything set,
* the final step is to re-run your analyses using bootstrap weights
* (you can do this all along but it's very slow to run)
* You may need to readjust your models if the results are quite
different

***** Redo all analyses using one of the svy commands:
*svy: tabulate
*svy: mean
*svy: regress
*svy: logit

* for means split by main IV:
svy, subpop (dumeverIPV): mean dumuseBasicANC_8
svy, subpop (dumNeverIPV): mean dumuseBasicANC_8
svy, subpop (dumeverIPV): mean dumDecisions
svy, subpop (dumNeverIPV): mean dumDecisions
svy, subpop (dumeverIPV): mean dumAge_15_24
svy, subpop (dumNeverIPV): mean dumAge_15_24
svy, subpop (dumeverIPV): mean dumAge_25_34
svy, subpop (dumNeverIPV): mean dumAge_25_34
svy, subpop (dumeverIPV): mean dumAge_35_49
svy, subpop (dumNeverIPV): mean dumAge_35_49

svy, subpop (dumeverIPV): mean dumrural
svy, subpop (dumNeverIPV): mean dumrural

svy, subpop (dumeverIPV): mean dumurban
svy, subpop (dumNeverIPV): mean dumurban

svy, subpop (dumeverIPV): mean dumeNOeduc_B
svy, subpop (dumNeverIPV): mean dumeNOeduc_B
svy, subpop (dumeverIPV): mean dumPRIMed_B
svy, subpop (dumNeverIPV): mean dumPRIMed_B
svy, subpop (dumeverIPV): mean dumSECedPlus
svy, subpop (dumNeverIPV): mean dumSECedPlus
```

```
svy, subpop (dumeverIPV): mean dumhusSECedPLUS
svy, subpop (dumNeverIPV): mean dumhusSECedPLUS
svy, subpop (dumeverIPV): mean dumhusedUnknown_B
svy, subpop (dumNeverIPV): mean dumhusedUnknown_B

svy, subpop (dumeverIPV): mean dumhusemp_b
svy, subpop (dumNeverIPV): mean dumhusemp_b

svy, subpop (dumeverIPV): mean dumdrink
svy, subpop (dumNeverIPV): mean dumdrink
svy, subpop (dumeverIPV): mean dumOneKid
svy, subpop (dumNeverIPV): mean dumOneKid
svy, subpop (dumeverIPV): mean dum2to4Kids
svy, subpop (dumNeverIPV): mean dum2to4Kids
svy, subpop (dumeverIPV): mean dum5plusKids
svy, subpop (dumNeverIPV): mean dum5plusKids
svy, subpop (dumeverIPV): mean dumNOTPaidorWork
svy, subpop (dumNeverIPV): mean dumNOTPaidorWork
svy, subpop (dumeverIPV): mean dumPaid
svy, subpop (dumNeverIPV): mean dumPaid
svy, subpop (dumeverIPV) : mean dumTradRelig
svy, subpop (dumNeverIPV): mean dumTradRelig
svy, subpop (dumeverIPV) : mean dumIslam
svy, subpop (dumNeverIPV): mean dumIslam
svy, subpop (dumeverIPV) : mean dumCatholic
svy, subpop (dumNeverIPV): mean dumCatholic
svy, subpop (dumeverIPV) : mean dumChristian
svy, subpop (dumNeverIPV): mean dumChristian
svy, subpop (dumeverIPV) : mean dumOther_Relig
svy, subpop (dumNeverIPV): mean dumOther_Relig
svy, subpop (dumeverIPV) : mean dumNORelig
svy, subpop (dumNeverIPV): mean dumNORelig
svy, subpop (dumeverIPV) : mean dumBeatJustified
svy, subpop (dumNeverIPV): mean dumBeatJustified

* for t-tests do as bivariate regressions by main IV
svy: logit dumuseBasicANC_8 dumeverIPV
svy: logit dumuseBasicANC_8 dumNeverIPV
svy: logit dumDecisions dumeverIPV
svy: logit dumNODecisions dumeverIPV
svy: logit dumAge_15_24 dumeverIPV
svy: logit dumAge_25_34 dumeverIPV
svy: logit dumAge_35_49 dumeverIPV
svy: logit dumrural dumeverIPV
svy: logit dumurban dumeverIPV

svy: logit dumeNOeduc_B dumeverIPV
svy: logit dumPRIMed_B dumeverIPV
```

```
svy: logit dumhusemp_b dumeverIPV

svy: logit dumdrink dumeverIPV
svy: logit dumPaid dumeverIPV
svy: logit dumNOTPaidorWork dumeverIPV
svy: logit dumOneKid  dumeverIPV
svy: logit dum2to4Kids dumeverIPV
svy: logit dum5plusKids dumeverIPV
svy: logit dumTradRelig dumeverIPV
svy: logit dumIslam  dumeverIPV
svy: logit dumCatholic  dumeverIPV
svy: logit dumChristian dumeverIPV
svy: logit dumOther_Relig  dumeverIPV
svy: logit dumNORelig dumeverIPV
svy: logit dumBeatJustified dumeverIPV


* REGRESSION ANALYSIS WITH WEIGHTED DATA
svy: logistic dumuseBasicANC_8 dumeverIPV dumDecisions dumAge_25_34
 dumAge_35_49 dumrural dumPRIMed_B dumSECedPlus dumpoorer dummiddle
 dumrich dumrichest dumworkingwoman dumPaid dumhusPRIMed_B
dumhusSECedPLUS dumhusedUnknown_B dumhusemp dumdrink dum2to4Kids
dum5plusKids  dumCatholic dumChristian dumTradRelig dumOther_Relig
dumNORelig dumBeatJustified

outreg2 using TakeJan_8.xls,  alpha (0.001, 0.01, 0.05) symbol(***,
 **, *) append sideway stats(coef se ci pval) eform
```
